# Advances in Modern Microsurgery

**DOI:** 10.3390/jcm13175284

**Published:** 2024-09-06

**Authors:** Oliver C. Thamm, Johannes Eschborn, Ruth C. Schäfer, Jeremias Schmidt

**Affiliations:** 1 Clinic of Plastic and Aesthetic Surgery, Helios Hospital Berlin-Buch, Schwanebecker Chaussee 50, 13125 Berlin, Germany; oliver.thamm@helios-gesundheit.de (O.C.T.); jm.eschborn@gmail.com (J.E.); 2 Clinic for Plastic and Reconstructive Surgery, Handsurgery, Burn Care Center, University Witten/Herdecke, Cologne-Merheim Medical Center, Ostmerheimer Straße 200, 51109 Cologne, Germany; 3 Clinic of Plastic, Reconstructive and Aesthetic Surgery, Helios Dr. Horst Schmidt Hospital Wiesbaden, Ludwig-Erhard-Straße 100, 65199 Wiesbaden, Germany; ruth.schaefer@helios-gesundheit.de

**Keywords:** robotic-assisted surgery, peripheral nerve reconstruction, microsurgery innovations, microsurgery, lymphatic surgery advances

## Abstract

**Background/Objectives**: Microsurgery employs techniques requiring optical magnification and specialized instruments to operate on small anatomical structures, including small vessels. These methods are integral to plastic surgery, enabling procedures such as free tissue transfer, nerve reconstruction, replantation, and lymphatic surgery. This paper explores the historical development, advancements, and current applications of microsurgery in plastic surgery. **Methods**: The databases MEDLINE (via PubMed) and Web of Science were selectively searched with the term “(((microsurgery) OR (advances)) OR (robotic)) OR (AI)) AND (((lymphatic surgery) OR (peripheral nerve surgery)) OR (allotransplantation))” and manually checked for relevance. Additionally, a supplementary search among the references of all publications included was performed. Articles were included that were published in English or German up to June 2024. **Results**: Modern microsurgical techniques have revolutionized plastic surgery, enabling precise tissue transfers, improved nerve reconstruction, and effective lymphedema treatments. The evolution of robotic-assisted surgery, with systems like da Vinci and MUSA, has enhanced precision and reduced operative times. Innovations in imaging, such as magnetic resonance (MR) lymphography and near-infrared fluorescence, have significantly improved surgical planning and outcomes. **Conclusions**: The continuous advancements in microsurgery, including supermicrosurgical techniques and robotic assistance, have significantly enhanced the capabilities and outcomes of plastic surgery. Future developments in AI and robotics promise further improvements in precision and efficiency, while new imaging modalities and surgical techniques expand the scope and success of microsurgical interventions.

## 1. Introduction

The term microsurgery encompasses techniques that require optical magnification and specialized micro-instruments to successfully operate on small anatomical structures, such as small vessels. These methods are employed across various surgical fields. In plastic surgery, microsurgical techniques are a crucial part of the reconstructive surgical spectrum and serve as the foundation for free autologous tissue transfer, peripheral nerve reconstruction, replantation and lymphatic surgery. The historical development of modern microsurgery is closely tied to advancements in microscopes and micro-instruments. In the 1950s and 1960s, with the introduction of coaxial illumination and motorized zoom, microsurgery was first clinically applied to perform limb replantation procedures via anastomosis of small-caliber arteries, veins, and peripheral nerves [1]. Dr. Chen and colleagues successfully replanted a completely amputated hand in 1963 in Shanghai [2].

In the 1970s, the first experimental tissue transfers were performed at various centers worldwide. The subsequent evolution in free tissue transfers during the 1980s and 1990s was driven by a better anatomical understanding of tissue perfusion. Taylor’s angiosome concept demonstrated specific vascular territories and their interconnections [3]. This led to numerous available flaps and donor regions for tissue transfer. Since the 1980s, microsurgery has revolutionized plastic surgery by enabling the transfer of various vascularized tissues, including skin, muscle, bone, and even composite tissue flaps such as complete joints. At the beginning of the 21st century, the first allotransplantations were reported. By 2006, Devauchelle and his colleagues successfully initiated the field of facial transplantation, considered the highest and most complex level of vascularized composite allotransplantation [4]. Patient selection remains ethically critical due to the need for lifelong immunosuppression despite the non-vital indication.

Today, the size of the structures no longer appears to be a limiting factor for further advances. In the last decade, supermicrosurgical techniques have been refined to perform anastomosis of lymphatics and blood vessels with diameters below 0.5 mm [5]. These techniques enable even more minimally invasive tissue transfer and a specific, tailored inset of tissue to suit the defect.

Performing supermicrosurgery requires special instruments, such as forceps with tip diameters of 0.1 mm and suture material sized 11-0 or 12-0. Utilizing these instruments demands specially trained motor skills and frequent practice to avoid unnecessary increases in operating time or severe complications.

Lymphatic surgery has recently evolved significantly due to advancements in supermicrosurgery and lymphatic imaging via MR lymphography and near-fluorescence angiography, making it the fastest-growing field in plastic surgery involving microsurgical skills. Arguably, the most substantial changes in the course of surgery, particularly microsurgery, are driven by the implementation of AI-operated robots.

## 2. Robotic Microsurgery

Since successful microsurgery depends on high-precision instruments in combination with fine motor skills, it is inevitable that robotics will play a dominant role in this field in the future. Currently, the main benefit of robotic-assisted surgery lies in the ability to operate on deep anatomical structures through small incisions. This has led to the routine integration of human-controlled semi-autonomous robots, such as the da Vinci system, in many clinics worldwide. Although plastic surgery primarily concerns superficial regions and open surgical approaches, there have been successful reports of intra-abdominal DIEP flap dissection for breast reconstruction using the da Vinci system. The greatest potential lies in the application of robots in microsurgery, particularly lymphatic surgery.

For selected indications, AI-powered autonomous robots are already considered superior to even highly trained surgeons in terms of tremor reduction and movement scaling, leading to a greater range of motion with increased precision [6]. Additionally, the surgeon can operate in an ergonomic position while viewing a 3D screen, which is an important factor during long, complex procedures.

After the first robotic-assisted microsurgical anastomosis in 2007 (da Vinci), the MUSA system (MicroSure, Eindhoven, The Netherlands) was introduced in 2014 as the first robot designed specifically for microsurgery. This unit is operated via a console with joysticks, allowing the surgeon to view a screen through a digital or hybrid microscope. The second CE-certified system is the Symani Surgical System (Medical Microinstruments, Inc., Wilmington, DE, USA), which is operated with free-moving joysticks designed to mimic actual microinstruments like forceps.

Regarding operating time, Barbon et al. demonstrated, in a case series involving 31 microanastomoses, an average operating time of 14.1 ± 4.3 min for the hand-sewn group compared to 25.3 ± 12.3 min for one robot-assisted anastomosis. Despite the time difference, they reported a steep learning curve with nearly similar times towards the end of the series [7]. Van Mulken et al. reported even shorter operative times using the MUSA robot compared to conventionally sutured lymphovenous anastomoses [8].

Further advancements in AI-powered enhanced intraoperative imaging, such as near-infrared cameras and ICG fluorescence to generate 3D images, promise significant improvements in visualization [9]. Currently, the costs pose a significant barrier to widespread clinical application, ranging between USD 400,000 and USD 650,000 for the complete MicroSure robotic system. Other systems incur even higher initial costs, such as USD 2 million for the da Vinci XI. Many authors believe that surgery will fundamentally change in the future with the implementation of robotics and AI, starting with ergonomic working positions, AI-enhanced perception of the operative field and anatomy, and even perception of the surgical environment [10].

A major drawback of robot-assisted surgery using joysticks is the lack of haptic feedback. For a surgeon, the feel of the tissue provides critical information, and the right tension is crucial for all kinds of dissections. In the future, this information is expected to be conveyed via so-called “e-skin” soft-surface robots and even enhanced beyond human perception through metabolic biosensing. Efforts are being made to harvest energy from the body via biofuels or respiration to create self-powered sensing tools for the next generation of medical devices [11]. Other areas of development include miniaturized robotic devices that enter the body through small incisions, such as for endovascular heart valve repair [12]. Semi-autonomous platforms used in clinics today still rely entirely on human control, while AI assists with tremor control or provides haptic feedback. The shift to fully autonomous AI-powered robotic surgery promises greater precision and efficiency than humans but also needs to overcome ethical and legal obstacles concerning liability.

## 3. Advances in Peripheral Nerve Surgery

Peripheral nerve injuries result in severe loss of function contributing to profound effects on mental and physical well-being of patients and therefore placing a significant burden on the patient and society [13,14]. Improved understanding and diagnostics of nerve injuries and their regeneration have increased surgical options. Routinely, a wide variety of nerve grafting, tendon transfer, and joint fusion techniques with variable evidence are available to improve function [15,16]. Furthermore, the focus of microsurgical reconstruction currently includes sensation and pain management of injured and reconstructed regions [17,18].

### 3.1. Advances in Diagnosis

In recent decades, the ease and availability of high-resolution nerve-imaging techniques has helped in defining pathologies, facilitated monitoring, and opened up new paths in surgery [19,20]. High-resolution ultrasound has become a handy tool not only utilized by the experienced diagnostician but also leading the surgeons hand. Rapid availability and the chance of intraoperative application enhances visibility and enables complex surgical planning. Improving the contrast of magnetic resonance imaging (MRI), magnetic resonance neurography (MRN) also provides a promising approach for detecting lesions. Still lacking clinical evidence, near-infrared nerve imaging constitutes a potentially interesting tool for intraoperative nerve visualization [21].

### 3.2. Advances of Nerve Bridging and Transfer

Increasing knowledge of axonal counts, fascicular topography, innervation patterns and clustering and location of nerves allows for specific designs in nerve reconstruction and transfer. While direct nerve suture, nerve grafting by autologous nerves, or processed nerve allografts still constitutes the most common reconstructive options, the variety of conduits such as collagen, silk fibroin, polylactic acid and chitosan tubes supersede muscle–vein autologous conduits [22]. Complications need to be considered; therefore, the autograft remains the gold standard for bridging nerve gaps. However, current research supports the use of processed nerve grafts for mixed motor and sensory nerve defects [23].

When direct coaptation or grafting fails or is anticipated to be insufficient, peripheral nerve transfers are utilized. Formulating a functional reconstructive plan is key to restoring motor and sensory function while minimizing donor site morbidity. The potential need for secondary tendon transfer options needs to be considered in upper and lower extremity reconstruction as well as in facial reanimation surgery [24,25].

**Figure 1:**
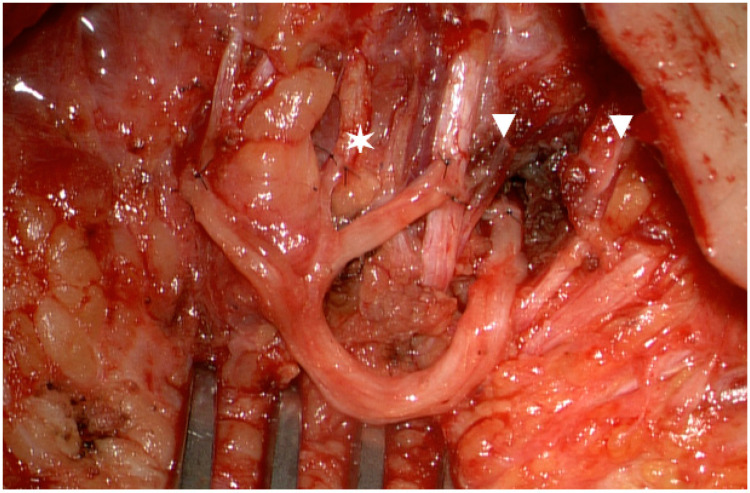
Sural nerve graft after interfascicular neurolysis coapted to the masseteric nerve, fascicles reaching a low zygomatic (left ✶), and two high buccal branches (▼).

In upper extremity reconstruction alongside traditional end-to-end transfer techniques, fascicular shift transfers of redundant motor branches with high axonal counts, which gain weight. For ‘supercharging’ intrinsic function of the hand in ulnar nerve injury, the distal branch of the anterior interosseous nerve is transferred to the side. The same principle of the babysitter procedure is utilized in facial nerve reinnervation by masseteric nerve branches (Figure 1) to allow for facial nerve regeneration [26,27].

Expanding the scope of nerve surgery, a combination of nerve transfers and technological advances in nerve and machine interfaces helps patients with amputations, spinal cord and brain lesions [18,28,29].

### 3.3. Advances in Sensitivity and Pain Management

The development of chronic pain and phantom pain by amputation or nerve injury is highly prevalent and greatly effects quality of life in the patient population. As a prevention or treatment option for neuroma development, target muscle reinnervation involves the nerve transfer of a sensory nerve to a motor branch of an adjacent muscle. Current data show highly favorable results in the formation of neuroma and the prevention of neuropathic pain when performed immediately during amputation [18,29]

Improvement in sensory and pain outcomes for neurotized free flaps in extremity and breast reconstruction can enhance the functionality of a weightbearing flap and quality of life for breast cancer survivors [17,30].

Timely restoration of motor function and sensitivity is a viable microsurgical reconstruction for patients with devastating nerve injuries. With ongoing refinements, results become more predictable and indications are increasing. Critical to success is the meticulous planning of desired function and expandable donor nerves as well as perioperative therapy and motor re-education. This section may be divided by subheadings. We provide a concise and precise description of the experimental results, their interpretation, as well as the experimental conclusions that can be drawn.

## 4. Free Microsurgical Tissue Transfer

For a long time, tissue transplantation was limited to free skin grafts (both split-thickness and full-thickness), local flaps, and pedicled regional or distant flaps. The survival of the transplanted tissue could only be ensured by local nutrition in a vascularized wound bed (skin grafts) or by translocation of an already existing vascular pedicle. Only microsurgery made it possible to transplant free tissue flaps by creating a new vascular connection to the flap pedicle at the recipient site. Microvascular surgery has developed steadily since the beginning of the 1960s. Jacobson and Suarez pioneered implementing the term microsurgery and described their vascular suturing technique using a microscope [31]. They were able to perform anastomoses of vessels with a diameter less than 3 mm in animals using magnifications from 25 up to 40. To perform these anastomoses, they had modified the operating microscope from the Zeiss–Littman version that was being used for auricular surgery [32]. Buncke and Schulz established principles and techniques of replantation and transplantation through numerous animal experiments and contributed to microsurgical training and education in this decade. After several successfully performed experiments by different surgeons, the first panel on microsurgery was held at the Annual Meeting of the American Society of Plastic and Reconstructive Surgeons in New York City in 1967.

In 1972, McLean and Buncke succeeded in transplanting the greater omentum to an extensive scalp defect in human with anastomoses of the gastroepiploic vessels to the superficial temporal vessels. The omentum was covered with a split-thickness skin graft [33]. Another milestone was the first transplantation of vascularized bone. In 1975, Taylor et al. performed the first free fibular transfer for reconstruction of a traumatic 12.5 cm defect of the tibia in a teenage male patient [34]. The first free microsurgical breast reconstruction with an adipocutaneous perforator flap from the abdomen pedicled on the deep inferior epigastric vessels (DIEP-flap) was published by Koshima and Soeda in 1989 [35]. To overcome the disadvantages of the pedicled TRAM-flap [36], they successfully operated on two patients with a DIEP-flap and showed that only one perforator is able to sufficiently perfuse a large adipocutaneous flap. These findings led to a paradigm shift, and DIEP flap reconstruction has become the gold standard in autologous breast reconstruction. Since then, several authors have described different flaps that included perforators of specific blood vessels (gluteal artery, thoracodorsal artery, profunda femoris artery) for perfusion of defined skin areas [37]. Wei et al. reported that a true perforator flap is a skin flap with its blood supply via musculocutaneous perforator vessels [38]. In modern plastic surgery, perforator flaps are a common standard for reconstructive procedures, since donor site morbidity is low. Microsurgery, commonly used for vessel anastomosis, flap harvesting, lymphedema, and nerve reconstruction, has traditionally relied on the operating microscope since its invention in the 1920s. Recent advances have introduced exoscopes as a valid alternative, with studies showing they are non-inferior to operating microscopes [39]. Despite initial concerns about image quality, newer exoscope models have addressed these issues, offering improved ergonomics and comparable performance in plastic surgery. The development of supermicrosurgery started in the 1990s. Technical advances in surgical microscopes and instruments allowed anastomoses of vessels and nerves with a diameter of 0.3 to 0.8 mm to be performed. Koshima was the first who presented cases of supermicrosurgical procedures in his lecture at the First International Course on Perforator Flaps in Belgium [40]. This new technique enabled the elevation of thin skin flaps like the superficial circumflex iliac artery perforator flap, lymph node flaps or even the anastomosis of lymphatic vessels.

Below, Table 1 and Table 2 are presented: the first summarizes the categories in which microsurgery and supermicrosurgery are commonly used, and the second provides an overview of the advancements and developments in microsurgery. 

**Figure 2:**
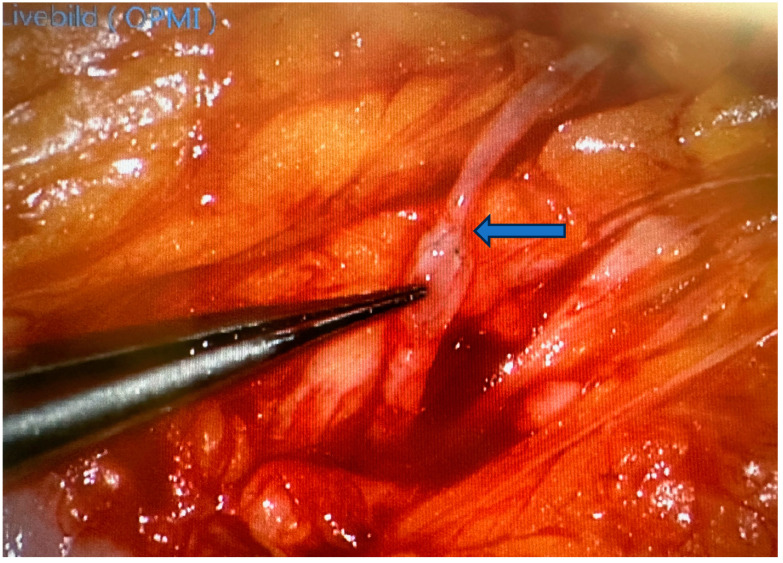
Supermicrosurgery: lymphatic reconstruction using end-to-end lymphovenous anastomosis with 11-0 suture material. Blue arrow showing the anastomosis.

**Figure 3:**
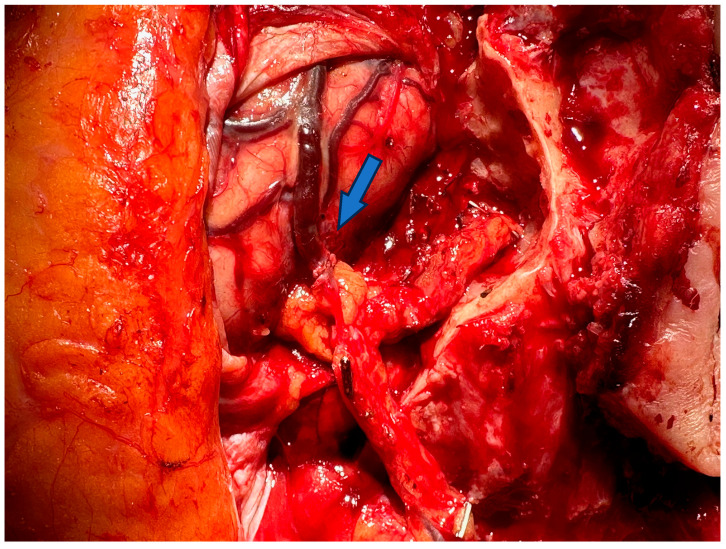
Microsurgery: vascular anastomosis in a neurosurgical–plastic surgery setting between the Vena Labee and the flap vessel, end to end, with 9-0 suture material. Blue arrow showing the anastomosis.

Following the tables are two pictures. Figure 2 is an example of a supermicrosurgical anastomosis between a vein and a lymphatic vessel in a lymphatic surgical procedure to treat a lymphatic fistula and Figure 3 shows a microsurgical anastomosis between the vena labee and the flap vein in reconstructive defect coverage. 

## 5. Replantation and Allotransplantation

One of the first clinical applications of microsurgery was the replantation of amputated limbs, starting with larger vessels at the proximal parts of the limbs. In 1962, Malt and McKhann successfully replanted the completely amputated arm of a 12-year-old boy including microvascular anastomoses of the axillary artery and two veins [41].

With advances in microsurgery in the 1960s, more peripheral amputations of the limb have been replanted. The first hand replantation at the level of the distal forearm was performed in China in 1963 [42]. One year later, Kleinert and Kasdan [43] successfully revascularized a partially severed thumb, while Komatsu and Tamai [44] successfully replanted a completely amputated thumb in 1965. In 1966, Chen accomplished a reconstruction of a thumb with a free second toe transfer [45].

In addition to providing a sufficient perfusion of the replant or transplant, another crucial step is the restoration of the functional structures and their neural innervation. In particular, when replanting fingers or transferring toes, a high level of expertise and outstanding manual skills are required, combined with specialized equipment. Nevertheless, the indication and secondary treatment also play a crucial role. Best outcomes after replantation are not solely related to the success of the microvascular anastomosis but also to the functional and aesthetic outcomes [46].

Composite tissue allografts (CTAs) or now called vascularized composite tissue allografts (VCAs) are one of the most challenging procedures in microsurgery and are still rare operations worldwide, only carried out in selected cases. VCAs are flaps that are composed of multiple different tissues but are transplanted together as a single unit. The first successful human CTA was an en-bloc digital flexor tendon mechanism by visionary North Carolina plastic surgeon Erle E. Peacock Jr. in 1957 [47]. However, an unsolved problem was the immunological rejection of allografts from genetically unrelated donors. The late 1950s and early 1960s brought the discovery of agents such as azathioprine, 6-mercaptopurine, and corticosteroids, which demonstrated prolonged graft survival in solid organ transplants in animal models [48,49]. Unfortunately, these results could not be reproduced in VCAs containing skin tissue. The introduction of cyclosporine A in 1976 revolutionized the allotransplantation by reducing acute rejection rates [50]. Survival times in VCAs also increased, but the skin parts were still rejected after a few weeks. For this reason, VCAs with skin have not been performed until the end of the 1990s, when new immunosuppressive drugs were available. Between 1998 and 1999, teams in Lyon (France), Louisville (USA), and Guangzhou (China) performed the first four successful hand transplantations using tacrolimus, mycophenolate mofetil, and corticosteroid combination therapy [51]. The first partial face transplantation was achieved in France by Dubernard and Devauchelle in 2005 [52]. The recipient died 11 years later due to cancer induced by immunosuppressive medication. The first full face transplant was performed in Barcelona (Spain) in 2010, including all intact functional and aesthetic units [53]. Meanwhile, more than 100 upper extremities and approximately 40 face transplants have been performed worldwide [54].

## 6. Lymphatic Surgery

The inception of lymphatic surgery was largely driven by the clinical need to address lymphedema, a chronic condition characterized by the accumulation of lymphatic fluid in the interstitial space, leading to significant swelling and associated morbidity. Conventional treatment modalities for lymphedema were largely palliative and provided limited efficacy, prompting the pursuit of more definitive surgical solutions.

In the 1960s, Dr. H. Shunji Yamada was among the pioneering surgeons who developed innovative techniques such as lymphaticovenous anastomosis (LVA), wherein lymphatic vessels are anastomosed to nearby veins to facilitate lymphatic drainage.

The field of lymphatic surgery experienced significant advancements with the advent of supermicrosurgery in the 1990s. Supermicrosurgery involves the manipulation of ultra-fine structures, often less than 0.5 mm in diameter, with even higher precision than traditional microsurgery. This breakthrough enabled surgeons to perform more refined and effective procedures, such as LVA and vascularized lymph node transfer (VLNT), which will be described later in this chapter.

Lymphedema is a chronic condition classified into primary (genetic) and secondary (more common) types, with the latter caused by diseases, treatments, infections, or injuries. Damage to the lymphatic system impairs fluid drainage, leading to swelling, typically in the lower extremities. This results in chronic inflammation, tissue fibrosis, and reduced immune function, increasing infection risk. Secondary lymphedema is especially prevalent in women due to oncological treatments, with an incidence of 0.13% to 2% in industrialized countries. A major breakthrough in lymphatic surgery has been the development of near-infrared fluorescence lymphangiography. This diagnostic method uses indocyanine green (ICG) administered intradermally/subdermally to visualize lymphatic vessels. It reveals the number, shape, and flow of lymphatic channels and identifies the stage of lymphedema through the dermal backflow pattern. Additionally, this technique is crucial for preoperative diagnostics, intraoperative navigation, reverse mapping, and postoperative monitoring of lymphatic reconstructive procedures [55,56,57,58]. As a complementary method for preoperative planning, MR lymphography can be used. This technique visualizes the anatomy of the lymphatic vessels and pathological changes with high diagnostic accuracy using gadolinium-based contrast agents. Through 3D reconstruction, it can precisely locate lymphatic pathways that are favorably positioned relative to an anastomosable vein. Using a coordinate system, exact surgical planning is possible. Additionally, MR lymphography can locate lymphatic vessels that are deeper than 1 cm and differentiate between vessels that are still patent and those that should not be redirected using LVA [59].

In principle, there is a distinction between reconstructive procedures and ablative (tissue-reducing) procedures. Reconstructive procedures aim to redirect or drain the accumulated lymph fluid by rerouting it into the venous system. Due to advancements in microsurgery toward supermicrosurgery and the availability of better imaging, finer instruments, and suture materials (11-0), it is now possible to anastomose lymphatic vessels with diameters as small as 0.3 mm to veins using end-to-end or end-to-side techniques. This surgical procedure is minimally invasive, as it involves very small skin incisions (about 2 cm) in the subcutaneous tissue. Using ICG or MR navigation, the lymphatic vessels are located and anastomosed to a vein using supermicrosurgical techniques. The second reconstructive procedure is the vascularized lymph node transfer. In these cases lymph nodes included in a tissue block are transferred as a free tissue graft to the affected extremity and then connected vascularly using microsurgical techniques.

In detail, lymphovenous anastomoses (LVA) are supermicrosurgical sutured connections between functional lymph collectors and venules. They facilitate the drainage of lymph fluid into the venous system before it reaches the damaged lymph node stations, reducing lymphedema in the affected anatomical region. The success of this technique depends on detecting functional lymph collectors and venules without significant reflux due to venous insufficiency or altered pressure conditions. The identification of functional lymph collectors before performing LVA can be achieved through the detection of the uptake of tracer dyes (ICG and/or lymph vessel-affine blue dyes like Patent Blue) and by controlling lymph flow via a stripping test or by observing visible lymph flow during the LVA procedure [60]. During the operation, ICG can be used with a portable near-infrared fluorescence camera or as an integrated module in the microscope.

To perform LVA, ultra-fine, special supra-microsurgical instruments are required, along with magnification aids in the microscope. Non-absorbable suture material of size 11-0 or 12-0 is preferred for physiological reconstructions in end-to-side, side-to-side, or side-to-end anastomosis. The procedure is usually performed with therapeutic intent, but in some cases, it can also be carried out prophylactically after surgical treatment of the lymph node stations (and thus before the onset of secondary lymphedema). It can be performed in anatomical (e.g., axillary) and extra-anatomical (distal) locations [61].

In addition to LVA, another supra-microsurgical treatment option is the use of VLNT. The VLNT consists of a free flap containing LN and surrounding adipose tissue that is transferred via microsurgical anastomosis to the recipient site. The perfused donor lymph node tissue in the recipient bed induces lymphangiogenesis mediated by growth factors, promoting the sprouting of blood and lymph vessels. Furthermore, the flap contains lymphatic capillaries capable of draining accumulated lymph fluid into the venous system through intranodal lymphovenous connections (shunts) within the lymph node itself. This results in immediate drainage via the anastomosed flap vein [62]. A special variant of the adipolymphatic VLNT is the adipo-lymphocutaneous VLNT with a skin island. This skin island can enlarge the contact area with the surrounding tissue, potentially improving the drainage capacity of the flap, restoring a pre-existing soft tissue defect, and/or serving as a monitoring island. This advanced lymphatic microsurgical technique can replace non-functional, lost lymph nodes (e.g., after surgical excision, infection, and/or radiation) with lymph nodes to reconnect to the existing lymphatic network through lymphangiogenesis, thereby increasing transport capacity and subsequently reducing the lymphatic load in the affected anatomical region [63,64].

A direct assessment of the patency of a lymphovenous anastomosis is significantly more challenging and not always as possible as conventional vascular anastomosis in microsurgery. Therefore, the success rates in supermicrosurgery are often a subject of discussion.

Various possible donor regions for lymph node transfer include the submental region, supraclavicular region, thoracodorsal region, and lateral inguinal region. Alternatively, with the help of abdominal surgeons, mesenteric VLNT can be harvested from the greater omentum (right gastroepiploic region) or jejunal mesentery. Free VLNT is considered the more invasive procedure among reconstructive techniques with the risk of donor site lymphedema. Therefore, reverse mapping of the donor region is considered mandatory to reduce donor morbidity (iatrogenic lymphedema). Particularly, the harvest of groin or axillary lymph nodes carries a risk for the development of donor site lymphedema [65,66]. The intra-abdominal, preferably mesenteric–jejunal harvest of VLNT is the latest donor region with the least experience [67,68]. However, the risk of donor site lymphedema is negligible, but the harvest remains the most invasive technique compared to all reconstructive procedures with potential visceral complications (scar hernia, ileus).

LVA can also be used as a therapy for treatment-resistant lymphoceles after operations in the lymph node regions. In this case, after locating the lymphatic vessel(s) feeding the lymphocele, the vessel is not closed but reconstructed through an LVA.

The further development of microsurgery towards supermicrosurgery has opened up new possibilities in the treatment of lymphoedema with LVA and VLNT. This development has been supported by ever finer instruments and the ability to visualize the lymphatic system functionally with ICG lymphangiography and high-resolution ultrasound. The cultivation of lymphatic tissue in the AV loop model is a visionary approach and reflects the current state of research.

## 7. Conclusions

Microsurgery, a field defined by operating on small anatomical structures with the aid of optical magnification and specific instruments, has significantly advanced over the past few decades. Initial breakthroughs in the 1950s and 1960s laid the foundation for the clinical application of microsurgical techniques in limb replantation. Subsequent decades saw the development of free tissue transfers and the refinement of techniques like anastomosis of small-caliber vessels and nerves.

The 1980s and 1990s marked a pivotal period with the introduction of the angiosome concept and the evolution of free tissue transfers, allowing for more precise and varied reconstructive options. Microsurgery further expanded with the advent of supermicrosurgical techniques, enabling the anastomosis of lymphatic vessels and blood vessels under 0.5 mm in diameter. These advancements facilitated more tailored reconstructions.

Robotics and AI have begun to play a crucial role in enhancing the precision and capabilities of microsurgery. Despite higher initial costs and the lack of haptic feedback, these technologies are likely to revolutionize surgical practices by improving precision and ergonomics.

In peripheral nerve surgery, advances in imaging and understanding of nerve anatomy have improved diagnostic capabilities and surgical outcomes. Techniques like nerve grafting and nerve transfers are now more refined, offering better functional recovery and pain management for patients with nerve injuries. Free microsurgical tissue transfer has evolved from basic skin grafts to complex procedures involving perforator flaps, which offer lower donor site morbidity and higher success rates.

Lymphatic surgery has also seen significant advancements, particularly with the use of near-infrared fluorescence lymphangiography and MR lymphography. These imaging techniques have improved the precision of procedures like lymphaticovenous anastomosis (LVA) and vascularized lymph node transfer (VLNT), offering treatment options for patients with lymphedema.

Overall, the continuous evolution of microsurgical techniques and the integration of advanced technologies like robotics and AI are set to further meliorate the precision, efficacy, and scope of surgical interventions, ultimately improving patient outcomes in various reconstructive procedures. Furthermore microsurgery will continue to develop as a result of tremor suppression by robotics, ever smaller access points, and completely new possibilities in AI-supported imaging (for example, using 3D images with superimposed ICG imaging on an exoscope). Tissue engineering is also expected to provide new approaches in microsurgery, when microsurgical transfer of lymphocytes cultivated in the AV loop model could be possible.

## Figures and Tables

**Table 1:** Categories in which microsurgery and supermicrosurgery are commonly used.

Category	Microsurgery	Supermicrosurgery
Vascular Anastomosis	Commonly used for vessels larger than 0.8 mm	Used for very small vessels (less than 0.8 mm)
Flap Reconstruction	Used for transferring tissue flaps	Used for thin, perforator-based flaps
Lymphedema Treatment	-	Lymphaticovenous anastomosis
Nerve Repair and Reconstruction	Nerve grafting and coaptation	Repair of very small nerve branches
Breast Reconstruction	DIEP, TRAM, and other large flap transfers	-
Hand Surgery	Replantation and composite tissue transfer	Replantation of distal fingers, fine vascular anastomosis
Facial Reanimation	Cross-facial nerve grafting	Supermicrosurgical nerve grafting, muscle transfers
Peripheral Nerve Surgery	Repair of larger peripheral nerves	Supermicrosurgical repair of small nerve branches

**Table 2:** Overview of the advancements and developments in microsurgery, including the years when these techniques were first applied and the vessel calibers.

Year	Advancement/Development	Vessel Caliber	Description
1921	Introduction of the first surgical microscope	~1.0 mm	Enabled more precise surgical procedures under magnification
1960s	Development of microsurgical instruments	0.5–1.0 mm	Specialized tools for handling tiny vessels and nerves
1964	First successful digital replantation	~1.0 mm	Marked the beginning of reconstructive microsurgery
1970s	Advent of microsurgical training programs	~0.8–1.0 mm	Formalized training improved the precision and success rates of microsurgery
1980s	Introduction of free tissue transfer (flap surgery)	0.3–1.0 mm	Allowed for complex reconstructions using tissue from distant body sites
1990s	Development of perforator flaps	0.5–1.0 mm	Advanced techniques for tissue transfer with minimal donor site morbidity
2000s	Emergence of supermicrosurgery	<0.8 mm	Enabled suturing of extremely small vessels and lymphatic vessels
2010s	Advancements in lymphaticovenular anastomosis (LVA)	<0.5 mm	Refined supermicrosurgical techniques for treating lymphedema
2020s	Introduction of robotic-assisted microsurgery	~0.5 mm and smaller	Enhanced precision and dexterity in microsurgical procedures using robotics

## Abbreviations

AI	Artificial intelligence
CE	Conformité Européenne (European Conformity)
CTA	Composite tissue allografts
MUSA	MicroSure surgical assistant
DIEP flap	Deep inferior epigastric perforator flap
MR	Magnetic resonance
LVA	Lymphovenous anastomosis
VCA	Vascularized composite tissue allografts
VLNT	Vascularized lymph node transfer
